# Comparative analysis of cyanobacterial superoxide dismutases to discriminate canonical forms

**DOI:** 10.1186/1471-2164-8-435

**Published:** 2007-11-27

**Authors:** Balakrishnan Priya, Jagadeesan Premanandh, Raman T Dhanalakshmi, Thangaraj Seethalakshmi, Lakshmanan Uma, Dharmar Prabaharan, Gopalakrishnan Subramanian

**Affiliations:** 1 National Facility for Marine Cyanobacteria (Sponsored by Department of Biotechnology, Government of India), Bharathidasan University, Tiruchirappalli – 620 024, India; 2 School of Physics, Bharathidasan University, Tiruchirappalli – 620 024, India

## Abstract

**Background:**

Superoxide dismutases (SOD) are ubiquitous metalloenzymes that catalyze the disproportion of superoxide to peroxide and molecular oxygen through alternate oxidation and reduction of their metal ions. In general, SODs are classified into four forms by their catalytic metals namely; FeSOD, MnSOD, Cu/ZnSOD and NiSOD. In addition, a cambialistic form that uses Fe/Mn in its active site also exists. Cyanobacteria, the oxygen evolving photosynthetic prokaryotes, produce reactive oxygen species that can damage cellular components leading to cell death. Thus, the co-evolution of an antioxidant system was necessary for the survival of photosynthetic organisms with SOD as the initial enzyme evolved to alleviate the toxic effect. Cyanobacteria represent the first oxygenic photoautotrophs and their SOD sequences available in the databases lack clear annotation. Hence, the present study focuses on structure and sequence pattern of subsets of cyanobacterial superoxide dismutases.

**Result:**

The sequence conservation and structural analysis of Fe (*Thermosynechococcus elongatus *BP1) and MnSOD (*Anabaena *sp. PCC7120) reveal the sharing of N and C terminal domains. At the C terminal domain, the metal binding motif in cyanoprokaryotes is DVWEHAYY while it is D-X-[WF]-E-H-[STA]-[FY]-[FY] in other pro- and eukaryotes. The cyanobacterial FeSOD differs from MnSOD at least in three ways *viz*. (i) FeSOD has a metal specific signature F184X_3_A188Q189_......._T280_......_F/Y303 while, in Mn it is R184X_3_G188G189_......_G280......W303, (ii) aspartate ligand forms a hydrogen bond from the active site with the outer sphere residue of W243 in Fe where as it is Q262 in MnSOD; and (iii) two unique lysine residues at positions 201 and 255 with a photosynthetic role, found only in FeSOD. Further, most of the cyanobacterial Mn metalloforms have a specific transmembrane hydrophobic pocket that distinguishes FeSOD from Mn isoform. Cyanobacterial Cu/ZnSOD has a copper domain and two different signatures G-F-H-[ILV]-H-x-[NGT]-[GPDA]-[SQK]-C and G-[GA]-G-G-[AEG]-R-[FIL]-[AG]-C-G, while Ni isoform has an nickel containing SOD domain containing a Ni-hook HCDGPCVYDPA.

**Conclusion:**

The present analysis unravels the ambiguity among cyanobacterial SOD isoforms. NiSOD is the only SOD found in lower forms; whereas, Fe and Mn occupy the higher orders of cyanobacteria. In conclusion, cyanobacteria harbor either Ni alone or a combination of Fe and Ni or Fe and Mn as their catalytic active metal while Cu/Zn is rare.

## Background

Superoxide dismutases (SODs, E.C. 1.15.1.1) are the superfamily of metalloenzymes that dismutases the highly toxic and reactive superoxide radical (O_2 _^-^, by-product of aerobic metabolism) through a cyclic oxidation-reduction ('*ping-pon*g') mechanism. As described by McCord and Fridovich [1], it is the first line of defense to alleviate oxidative stress virtually in all living organisms that survive in oxic environment.

The evolutionary trajectory has favored SOD as a ubiquitous enzyme in multiple forms within a single organism or cell, indicating a fail-safe redundancy that emphasizes the importance of this family of enzymes against reactive oxygen species (ROS). Based on metal cofactors, four known (canonical) isoforms *viz*., iron (Fe), manganese (Mn), copper/zinc (Cu/Zn) and nickel (Ni) SODs have been identified. In general, SODs have a strict metal binding specificity for enzymatic activities with the exception of a class of enzymes which show enzymatic activity regardless of whether Fe or Mn is bound at the active site; these are known as cambialistic forms [2-5].

Cyanoprokaryotes are oxygen evolving photosynthetic organisms occupying a crucial position between pro- and eukaryotes. They are considered to be primeval having evolved about 3.2 billion years ago [6]. In addition, they succeeded in linking photosynthetic electron flow from water as the photoreductant through an oxygen-evolving complex at the high-potential side of the newly elaborated photosystem II, which is thought to have originated from a uniform primordial photosystem by gene duplication [7]. The resultant tandem operation of two photosystems is now known as oxygenic or plant-type photosynthesis [8]. This marked the turning point in the evolution of earth, opening up the era of an aerobic, oxygen-containing biosphere and SOD is found to play a critical role in mitigating the toxic effect of superoxide ion. The first implication on the protective role of cyanobacterial SOD in photo-oxidative damage was shown in *Anacystis nidulans *[9]. Subsequently, several studies on protective role of SODs of cyanobacteria in response to various physiological processes/stresses like photosynthesis [10], desiccation [11,12], chilling [13], nitrogen starvation [14] and with azo dyes (unpublished) have been reported.

Metal preferences in Fe and MnSODs have been well documented in both pro- and eukaryotic forms [15-17]. However, no information is available on distinguishing the canonical isoforms of cyanobacteria. Hence, the present study focuses on structure and sequence pattern of subsets of cyanobacterial SODs to explore the possibility of solving the ambiguity.

## Results and Discussion

For the survival of cyanobacteria with oxygenic photosynthesis, the selection pressure led to the evolution of SODs as the first antioxidant arsenal against nascent oxygen species. Studies on cyanobacterial SODs would serve as a window into the past and present evolutionary events of these primitive phototrophs.

**Figure 1:**
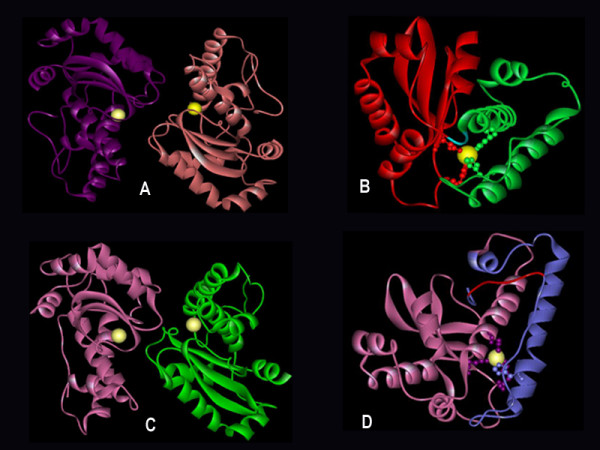
**Structure of Fe and MnSOD**. Structures are visualized using WebLab ViewerLite 4.2 software. Catalytically essential aspartate or histidine residues are represented in ball and stick mode binding the active metal (yellow) is shown to identify the location of the active site. Protein database codes are given in parentheses: (i) FeSOD (PDB 1gv3); (ii) MnSOD (PDB 1my6). (A) FeSOD of *T.elongatus *BP-1 dimers are distinguished by colour (violet and slate), and structures are represented with the active site (yellow) of subunit. (B) Monomeric subunit of FeSOD represents an N terminal (green) and a C- terminal (red). Similarly (C) represents dimer structure of *Anabaena *sp. MnSOD in pink and green with active site highlighted in yellow. (D) Monomeric MnSOD showing the N-terminal residues in blue and C-terminal in pink with metal binding ligands. The transmembrane hydrophobic pocket specific for MnSOD is highlighted in red (D).

On comparison, the canonical isoforms of SOD, Fe and MnSOD's are structurally distinct from Cu/Zn and NiSOD. Both Fe and MnSOD are typically homodimers or tetramers (Fig 1A,C) sharing identical metal chelating residues at the active site with a high degree of sequence and structural homology except for slight differences in amino acid residues. For instance, the amino acid range in cyanobacterial FeSOD is 199–229 residues with a molecular weight of 21–25 KDa, whereas in MnSOD, it is 200–316 amino acids with a molecular weight of 22–34 KDa.

**Figure 2:**
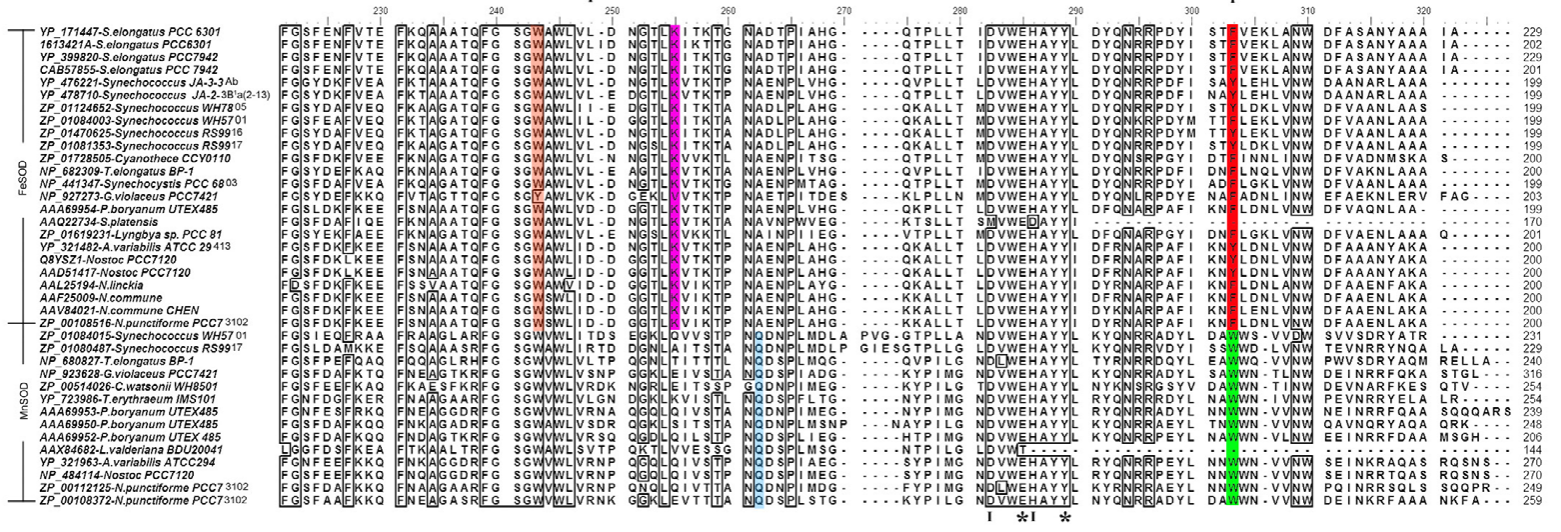
This figure shows the lower quartile of protein sequence alignment of Fe and MnSODs in cyanobacteria. The highly conserved metal specific residues are highlighted in red for Fe and green for MnSODs. Residues involved in outer sphere hydrogen bonding for Mn is highlighted in cyan and for Fe in orange. For FeSOD, the lysine residues involved in photosynthetic context is shown in pink. The active site residues are marked as **I **and the dimer residues are represented by .

Both SODs revealed a common topology with all α N-terminal (Pfam:PF00081) and a α/β C terminal domains (Pfam:PF02777) (Fig 1B,D). The sequence pattern for Fe and MnSODs of eukaryotes and other non-cyanobacterial prokaryotes is D-X-[WF]-E-H-[STA]-[FY]-[FY] [18]; whereas, the analysis of the sequence conservation in cyanobacteria (based on available data) showed a specific motif DVWEHAYY [D282-Y289, based on Fig 2]. This motif extends between the second α-helix and the first β-sheet of the C-terminal domain in both the SOD's. The highly conserved residues aspartate D282 and histidine H286, a constituent of the motif are the metal binding ligands. In addition, glutamic acid E285 and tyrosine Y289 form a dimer surface spanning the interface and bridging the active sites between the opposite halves of each subunit, see Figure 2 (For full image, please see Additional file 1).

**Figure 3:**
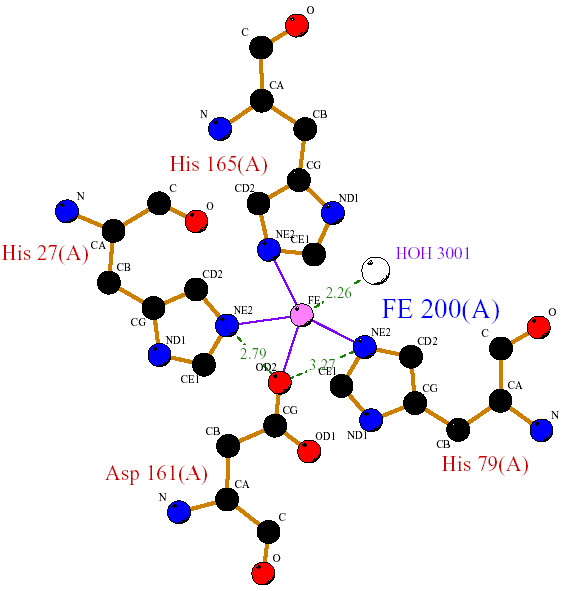
The active site residues of Fe Superoxide dismutase of *Thermosynechococcus elonagtus*.

**Figure 4:**
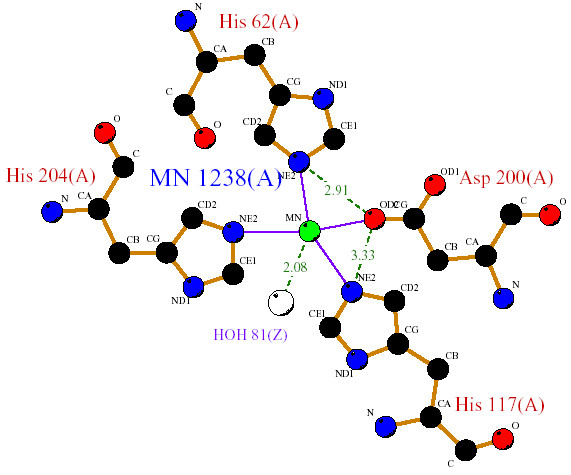
The active site residues of Mn Superoxide dismutase of *Anabaena sp*.

Structural analysis of available cyanobacterial Fe and MnSODs, confirms that both share a similar active site (i.e., metal ion) being coordinated in the respective isoform by three histidine and an aspartate residue with a ligating solvent molecule (water or OH), a five coordinated trigonal bipyramidal geometry. In *Thermosynechococcus elongatus *(PDB code 1my6); the Fe ion is coordinated by the carboxylate oxygen (Oδ2) of D161 with the amino group (Nε2) of H79, 27, 165 along with the oxygen atom of the water molecule. The hydrogen bonding distance between Oδ2 (D161) and Nε2 (H27 and H79) is 2.79Å and 3.27Å respectively (Table 1). In case of *Anabaena *sp (PDB code: 1gv3), the Mn is coordinated by Nε2 of H117, 204, 62 and Oδ2 of D200. The hydrogen bonding between Oδ2 (D200) and Nε2 (H62 and H117) is 2.19Å and 3.33Å respectively. These hydrogen bonds are involved in stabilizing the orientation of the ligand residues in MnSOD [8]. The observed contact surface area (31–35 Å^2^) between the side chain aspartate oxygen atom (Oδ2) and histidine (Nε2) implies that the metal coordination ligands in the exposed region may perhaps tune the redox potential (Fig 3, 4).

The motif and metal binding sites of Fe and Mn isoforms appear to exhibit similar function. However, the sequence alignment and structural analysis reveal their possible discrimination by three traits to specifically differentiate Fe and Mn isoforms (Table 1 Additional file 1).

**Table 1:** Discriminatory key to classify indecisive isoforms.

**Characteristics**	**FeSOD**	**MnSOD**
Metal specificity	Fe	Mn
Amino acid length	199–229	200–316
Theoretical molecular weight	21–25 KDa	22–34 KDa
No. of a helix*	13	14
No. of b strand*	3	3
Domains	N & C terminal	N & C terminal
Motif	DVWEHAYY	DVWEHAYY
Active site residues*	Fig 3	Fig 4
Structurally highly conserved metal specific residues	F_184_XXXA_188_Q_189......._T_280......_F/Y_303_	R_184_XXXG_188_G_189......._G_280......_W_303_
Conserved residue with photosynthetic role	K87, K139	None
Transmembrane hydrophobic pocket	Absent	Present

* – Based on the structural analysis of MnSOD of *Anabaena *sp. (PDB No: 1gv3) and FeSOD of *Thermosynechococcus elongatus *BP-1 (PDB No: 1my6)

**Figure 5:**
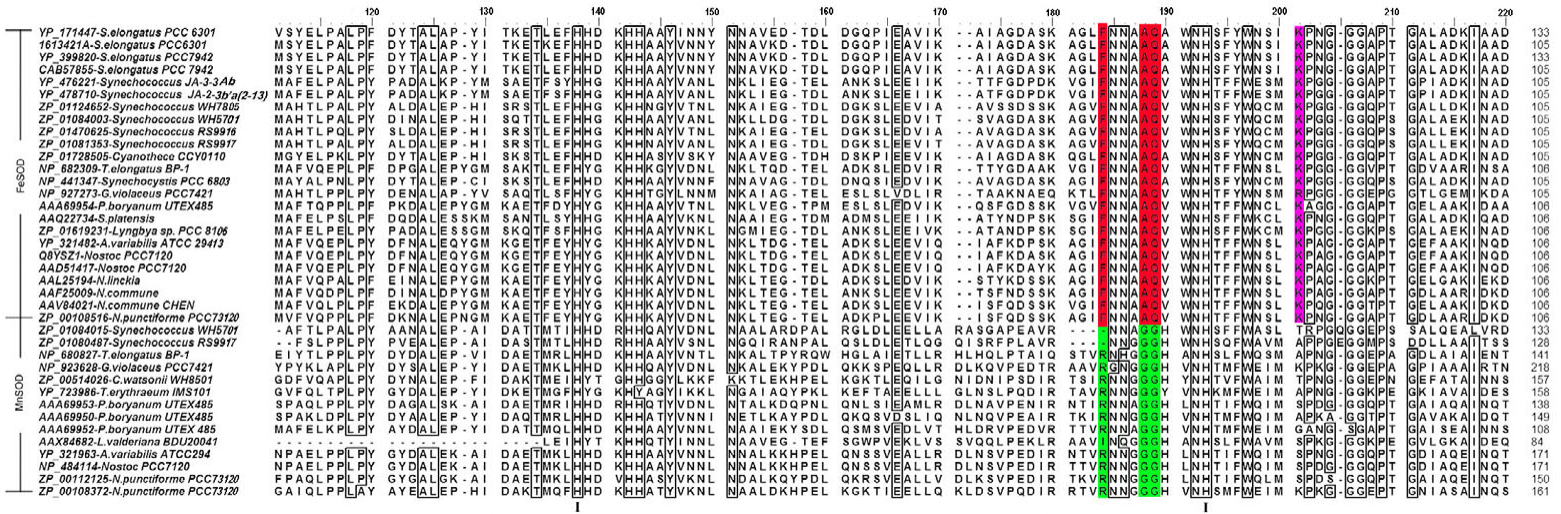
This figure shows the second quartile of protein sequence alignment of Fe and MnSODs in cyanobacteria. For full image, please see Additional file 1. The conserved aminoacid signature for Fe and MnSODs are highlighted in red and green respectively. Lysine residues of FeSOD involved in photosynthetic context is depicted in pink. The active site residues are labeled as **I**.

First, is the change in conserved amino acid signature F184X_3_A188Q189_......._T280_......_F/Y303 in Fe being replaced by R184X_3_G188G189_......._G280_......_W303 in MnSOD (see Figures 2 and 5).

The second notable feature is related to the metal bound solvent molecule that serves as a hydrogen bond to the non-coordinated oxygen of the carbonyl group of the aspartate ligand accepting a hydrogen bond from an outer sphere residue [19]. In MnSOD, it is glutamine Q262 (Fig 2) arising from the end of the β_2_-strand and H _9 _in the C-terminal domain, while in FeSOD, it is tryptophan W243 arising from the middle of the sequence (within the β_1_) in the C-terminal domain. In the case of cambialistic Fe/MnSOD metalloform reported in archaea (*Pyrobaculum aerophilum*) [19], the outer-sphere H-bonding residue is histidine. This residue plays a major role in altering the solvent interaction with the active site metal ion in cambialistic Fe/Mn SOD isoform [19]. The sequence analysis of cyanobacterial SODs showed the absence of this histidine residue which probably suggests the absence of cambialistic forms in cyanobacteria. Vance and Miller [20] reported that the most highly conserved residues glutamine Q262 in Mn and Q189 of FeSOD forms the outer sphere hydrogen-bond network exerts a large influence on redox midpoint potential tuning for catalytic activity of SOD's.

The third difference is the presence of two lysine residues, K201 and 255 in FeSOD but not in MnSOD (Fig 2 and 5). These residues seem to be unique and function specific to cyanobacteria among prokaryotes [21]. K201 lines a small pit at the surface of the *T. elongatus *and of higher plants FeSOD, formed by the loop P202-G203-G204 connecting N and C terminal domains. Likewise, K255 is restricted only to cyanobacteria, indicating its importance in the photosynthetic context [21].

**Figure 6:**
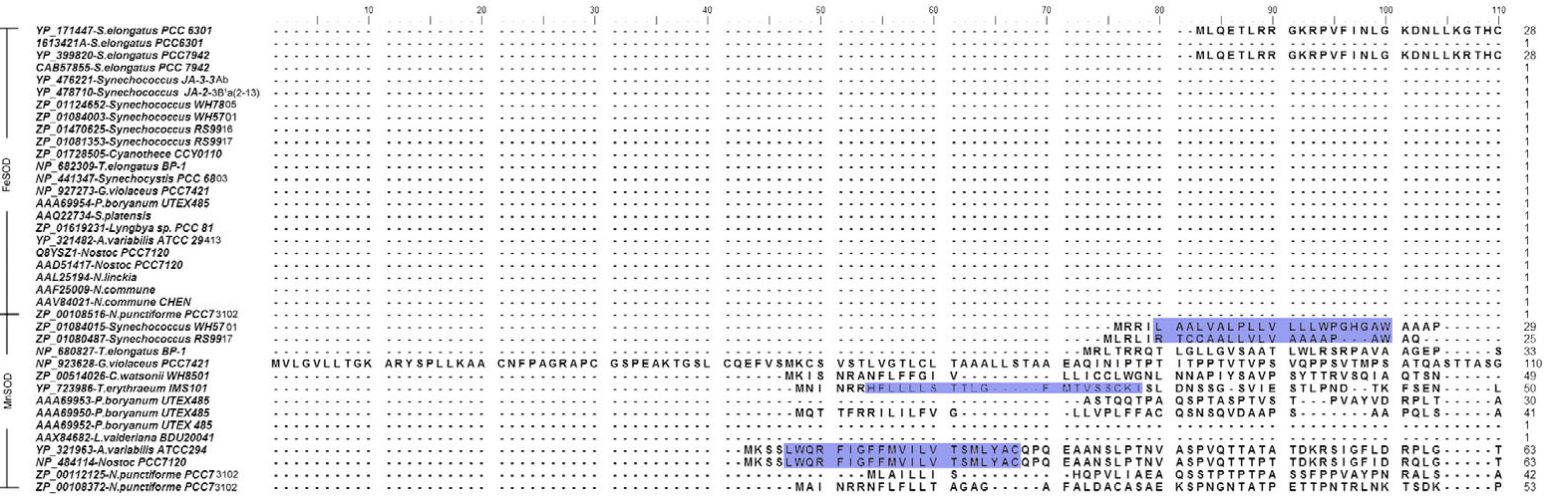
This figure shows the upper quartile of protein sequence alignment of Fe and MnSODs in cyanobacteria. For full image, please see Additional file 1. Transmembrane hydrophobic pocket specific for membrane binding in MnSOD at the N-terminal region is highlighted in violet.

Cyanobacterial MnSOD is the only SOD to be membrane anchored by transmembrane helix [22]. The factor that determines localization of MnSOD is found to span the N terminal which is a hydrophobic transmembrane helix (Fig 1D, 6). The cyanobacterial representatives such as (*Synechococcus *sp. WH5701 (EAQ76095), *Synechococcus *sp. RS9917 (EAQ68777), *Trichodesmium erythraeum *IMS101 (EAO27349), *Anabaena variabilis *ATCC29413 (ABA21068) and *Nostoc *sp. PCC7120 (BAB77594)) clearly corroborate this (Fig 6).

**Figure 7:**
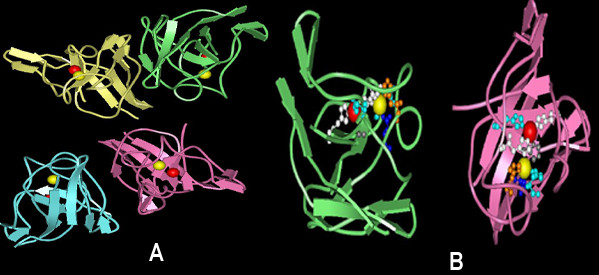
**Representative structure of *Salmonella typhimurium *Cu/Zn superoxide dismutase**. (a) Tetrameric subunits of Cu/ZnSOD. Chain A coded in green, B in pink, C in yellow and D in cyan. (b) Crystallographic structure of functional *S. typhimurium *Cu/ZnSOD (PDB 1eqw) subunit is represented to highlight the active site residues in ball and stick mode visualized using WebLab ViewerLite 4.2 software.

**Figure 8:**
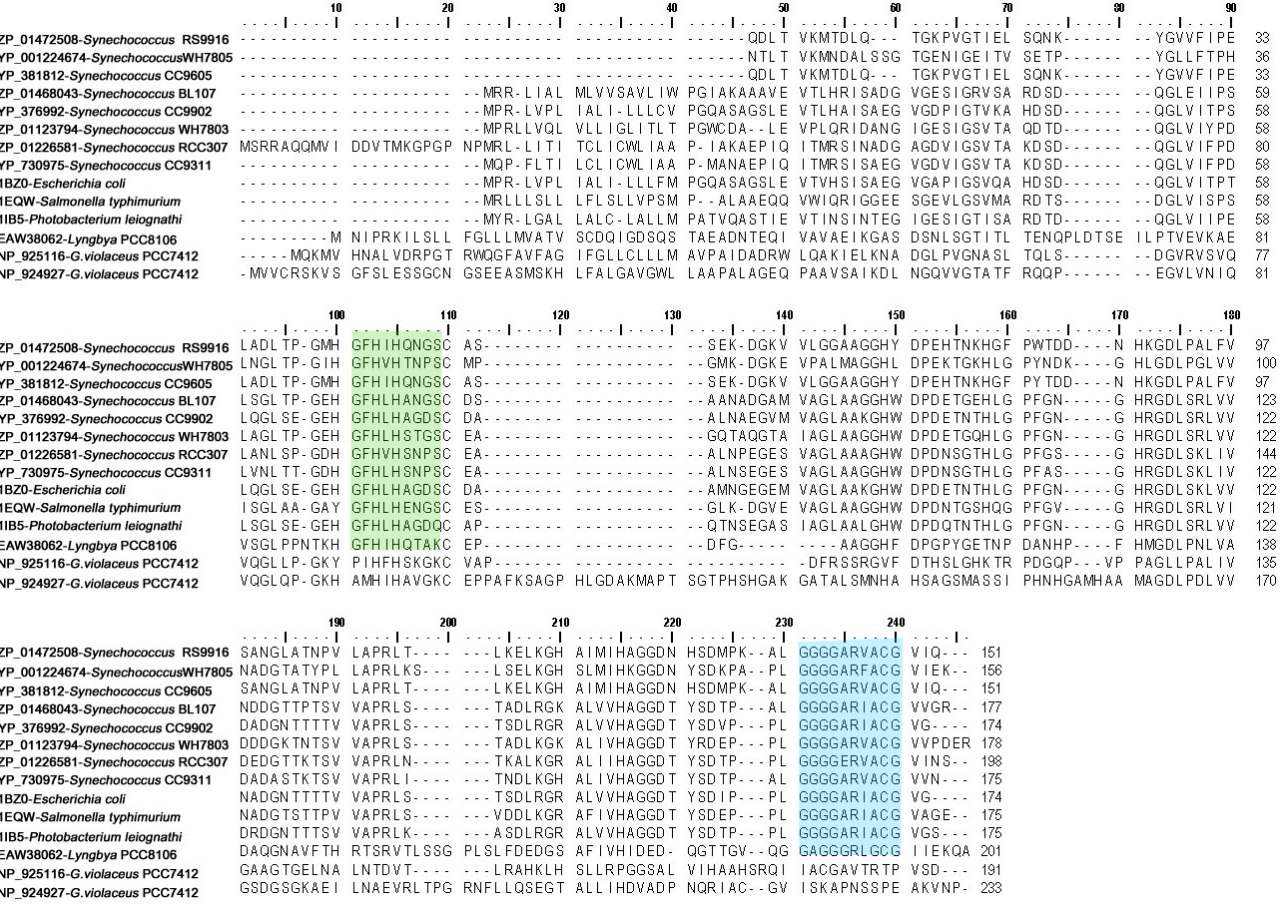
**Sequence alignment of cyanobacterial copper zinc superoxide dismutase with bacterial representatives**. Alignment was carried out using Clustal W of BioEdit Package (v.7.0.5) [28]. The active site Cu residues are marked as  and Zn in #. The signature 1 residues are highlighted in green and signature 2 in blue.

Cyanobacterial Cu/ZnSOD isoform bears no resemblance to Fe or Mn or Ni isoform in relation to its primary and tertiary structure. The theoretical molecular weight ranges between 16–23 KDa with an amino acid length of 174–233 residues. Further, study on amino acid composition illustrates that it is rich in Gly (11–16%) forming eight β-sheets (Fig 7A) accredited to be involved in conformation [23] and stability in repeated freeze/thaw cycles and prolonged refrigeration [9]. These isoforms in general have a copper containing domain (Pfam:PF00080) with two different signatures. The first is G-F-H-[ILV]-H-x-[NGT]-[GPDA]-[SQK]-C where the conserved histidine is involved in copper binding, and the second being G-[GA]-G-G-[AEG]-R-[FIL]-[AG]-C-G where C is involved in disulfide bonding (Fig 8). *G. violaceus *SOD (NP_925116, NP_924927) annotated as 'similar to SOD' contains only copper binding domain and both the signatures are absent. Further confirmation requires additional structural data. Each monomer is comprised of a binuclear metal centre with one Cu and one Zn atom. The noticeable β parallel fold of cyanobacterial Cu/Zn isoform mimics the structure of *Salmonella typhimurium *Cu/ZnSOD [24] (Fig 7B). The catalytic coordination sphere of Cu^2+ ^ion is by Nδ1 of H103, Nε2 of H105, H147 and H215 and Zn^2+ ^by Nδ1 of three H147, 157, 171 and Oδ1 of one D174 (Fig 8). Besides this, structural comparison designates the two specific hydrogen bonds between the Zn^2+ ^coordinating residues D174-Oδ1... H157-Nδ1 (3.25 Å) and D174-Oδ1... H171-Nε1 (3.18 Å) to ligand stability.

**Figure 9:**
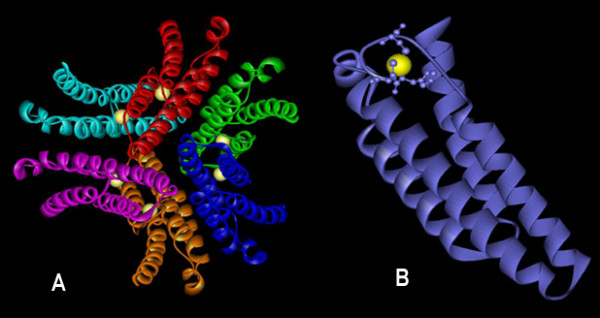
**Schematic view of representative NiSOD subunit and hexameric structure of *Streptomyces coelicolor *[PDB 1t6u]**. (a) NiSOD biological unit is a hexameric assembly of 4-helix bundles (b) NiSOD subunit with metal binding hook labels at the end of helix-1 along with the metal shaded in yellow is represented by ball and stick mode as visualized in WebLab ViewerLite 4.2 software.

The fourth canonical form NiSOD is a hexamer (Fig 9A) found only in cyanobacteria [25] and *Streptomyces *[26,27] with amino acids ranging from 140–163 and molecular weight between 15–18 KDa. Analysis of available sequences and complete genome sequences revealed that, unicellular *Prochlorococcus *forms possess only NiSOD, whereas, multicellular filamentous heterocystous and heterotrichous forms lacks this isoform (Table 2). The key for the ubiquity of NiSOD in *Prochlorococcus *may be due to the primitive photosynthetic machinery and its smallest genome size (between 1669–2434 Kb) by gene rearrangement or loss to maximize the energy economy [28]. The sequence conservation, motif with eleven-residues (HCDGPCVYDPA) in N-terminal region of Ni-hook, along with a nickel containing SOD domain (Pfam:PF09055) forms an unique pattern to identify cyanobacterial NiSOD. Cyanobacterial NiSODs seem to have an assembly of four alpha helices bundle with a short connecting alpha helix, as that of *Streptomyces sp*. (Fig 9B). The catalytic Ni ion of cyanobacteria is very much analogous to the reported square planar active center with thiolate (C2, based on 1t6u), backbone nitrogen (H1 and C6) ligands and of square pyramidal Ni (II) with an added axial His_1 _side chain of *Streptomyces sp*. [29].

**Table 2:** Annotation of cyanobacterial superoxide dismutases based on sequence and structure conservation.

**Organisms**	**Accession no**	**Sequence length**	**Type of SOD in Database**	**Confirmed isoform from our study**
*Prochlorococcus marinus AS9601*	YP_001009883	157	putative Ni	**NiSOD**
*Prochlorococcus marinus CCMP1986*	NP_893411	156	putative Ni	**NiSOD**
*Prochlorococcus marinus CCMP1375*	NP_875759	157	Ni	**NiSOD**
*Prochlorococcus marinus MIT 9301*	YP_00109170	157	putative Ni	**NiSOD**
*Prochlorococcus marinus MIT 9303*	YP_001017980	164	putative Ni	**NiSOD**
*Prochlorococcus marinus MIT 9211*	ZP_01004940	140	Ni	**NiSOD**
*Prochlorococcus marinus MIT 9312*	YP_397886	157	putative Ni	**NiSOD**
*Prochlorococcus marinus MIT 9313*	NP_894173	157	putative Ni	**NiSOD**
*Prochlorococcus marinus MIT 9515*	YP_001011769	157	putative Ni	**NiSOD**
*Prochlorococcus marinus NATL1A*	YP_0010155334	163	putative Ni	**NiSOD**
*Prochlorococcus marinus NATL2A*	YP_292055	163	putative Ni	**NiSOD**
*Synechococcus sp. WH 8102*	NP_897719	157	putative Ni	**NiSOD**
*Synechococcus sp. BL107*	ZP_01469600	157	putative Ni	**NiSOD**
	ZP_01468043	198	putative SOD	**Cu/ZnSOD**
*Synechococcus sp. CC9605*	YP_381196	157	putative Ni	**NiSOD**
	YP_381812	178	SOD precursor (Cu-Zn)	**Cu/ZnSOD**
*Synechococcus sp. CC9311*	YP_729969	175	Cu/Zn	**Cu/ZnSOD**
	YP_730975	155	Ni	**NiSOD**
*Synechococcus sp. CC9902*	YP_376992	175	putative SOD	**Cu/ZnSOD**
*Crocosphaera watsonii WH 8501*	ZP_00517273	159	Hypothetical protein	**NiSOD**
	ZP_00514026	254	SOD	**MnSOD**
*Synechococcus elogatus PCC 6301*	YP_171447	229	SOD	**FeSOD**
	1613421A	202	SOD	**FeSOD**
*Synechococcus elogatus PCC 7942*	YP_399820	229	SOD	**FeSOD**
	CAB57855	201	SOD	**FeSOD**
*Synechococcus sp. JA-3-3Ab*	YP_476221	199	Fe	**FeSOD**
*Synechococcus sp. JA-2-3B'a(2–13)*	YP_478710	199	Fe	**FeSOD**
*Synechococcus sp. WH 7805*	ZP_01124652	199	SOD	**FeSOD**
	ZP_01123794	174	putative SOD	**Cu/ZnSOD**
*Synechococcus sp. WH 5701*	ZP_01084003	199	SOD	**FeSOD**
	ZP_01084015	231	Mn	**MnSOD**
*Synechococcus sp. RS9916*	ZP_01470625	199	SOD	**FeSOD**
	ZP_01472508	177	SOD precursor (Cu-Zn)	**Cu/ZnSOD**
*Gloeobacter violaceus PCC 7421*	NP_927273	203	SOD	**FeSOD**
	NP_923628	316	SOD	**MnSOD**
	NP_924927	233	similar to SOD	NA*
	NP_925116	191	similar to SOD	NA*
*Synechococcus sp. RS9917*	ZP_01081353	199	SOD	**FeSOD**
	ZP_01080487	229	SOD	**MnSOD**
*Cyanothece sp. CCY0110*	ZP_01728505	200	SOD	**FeSOD**
*Thermosyncehococcus elongatus BP-1*	NP_682309	200	SOD	**FeSOD**
	NP_680827	240	SOD	**MnSOD**
*Lyngbya sp. PCC8106*	ZP_0169885	201	SOD	**Cu/ZnSOD**
	ZP_01619231	201	SOD	**FeSOD**
*Trichodesmium erythraeum IMS101*	YP_723986	254	SOD	**MnSOD**
	YP_720765	159	putative Ni	**NiSOD**
*Synechocystis sp. PCC 6803*	NP_441347	199	Fe	**FeSOD**
*Spirulina platensis*	AAQ22734	170	Fe	**FeSOD**
*Plectonema boryanum UTEX 485*	AAA69954	199	Fe	**FeSOD**
	AAA69953	239	superoxide dismutase [Mn] precursor	**MnSOD**
	AAA69950	248		**MnSOD**
	AAA69952	206		**MnSOD**
*Leptolyngbya valderiana BDU20041*	AAX84682	144	Mn	**MnSOD**
*Nostoc punctiforme PCC 73102*	ZP_00108516	200	SOD	**FeSOD**
	ZP_00112125	249	SOD	**MnSOD**
	ZP_00108372	259	SOD	**MnSOD**
*Nostoc sp. PCC 7120*	Q8YSZ1	200	Fe	**FeSOD**
	AAD51417	200	Fe	**FeSOD**
	NP_484114	270	SOD	**MnSOD**
*Anabaena variabilis ATCC 29413*	YP_321482	200	Mn/Fe	**FeSOD**
	YP_321963	270	Mn/Fe	**MnSOD**
*Nostoc linckia*	AAL25194	200	SOD	**FeSOD**
*Nostoc commune*	AAF25009	200	SOD	**FeSOD**
*Nostoc commune CHEN*	AAV84021	200	Fe	**FeSOD**

* Not Assignable (NA)

## Conclusion

The analysis is based on 64 cyanobacterial SODs available to date in public databases. Among them 2 are described as Fe/Mn, 4 as Cu/Zn and Mn precursor, 16 as putative NiSOD, 11 annotated as Fe, Mn and Cu/Zn isoforms, 29 as possible/putative SOD and 2 as hypothetical proteins.

Thus the present study resolves the incompletely annotated SODs among cyanobacteria (Table 2). Further, 64 cyanobacterial SOD sequences are clearly categorized into 17 NiSOD, 7 Cu/ZnSOD, 24 FeSOD and 14 MnSOD genes, 2 non assignable as they require further structural data. The strict metal specificity, precise sequence and structure among the metalloforms led to discriminate Mn and FeSOD (Table 1). The highly homologous Fe and MnSODs shares a metal binding motif DVWEHAYY without any variation, compared to D-X-[WF]-E-H-[STA]-[FY]-[FY] found in other pro – and eukaryotes.

The whole genome sequences analyses of cyanobacteria reveal that the primitive unicellular *Prochlorococcus *with simple photosynthetic apparatus possesses only NiSOD. The more evolved middle order forms of cyanobacteria posses a combination of Fe and Ni or Fe and Mn SODs. The most evolved filamentous, heterotrichous and heterocystous forms predominantly have only Fe and Mn metalloforms. However, CuZn also occurs rarely (Table 2).

## Methods

The non-redundant database of protein sequences (National center for Biotechnology Information, NIH, Bethesda) were retrieved using the PHI-BLAST [30] search tool using BLOSOM 62 matrix with gap penalities (Existence – 11 and Extension – 1) with a threshold value of 0.005 and optimal limit for cyanobacteria. The query sequence used were *Synechococcus *sp. JA-3-3Ab with Expasy-PROSITE pattern D-x-[WF]-E-H-[STA]-[FY]2 for Fe/MnSOD; *Synechococcus *sp. RSS9916 with signature 1 [GA]-[IMFAT]-H-[LIVF]-H-{S}-x-[GP]-[SDG]-x-[STAGDE] and signature 2 (G-[GNHD]-[SGA]-[GR]-x-R-x-[SGAWRV]-C-X(2)-[IV]) for Cu/ZnSOD. In addition, the individual sequences of all the SOD metalloforms were also manually retrieved from public databases (NCBI, KEGG). Identical sequences from the same organism were removed manually. *Intoto*, 64 sequences representing 24 complete genomes and individual submissions obtained are listed in Table 2 together with the accession numbers and the organisms. Identification of domains associated with SOD proteins were realized using NCBI Conserved Domain Search and Pfam servers

The secondary structure consensus was carried out using nnPREDICT [31] and JPRED [32] for each protein to refine the multiple sequence alignment. Multiple alignments for cyanobacterial Fe and MnSODs; and Cu/ZnSOD sequences were generated using the Clustal W (neighbor-joining) of BioEdit V.7.0.5 [33] program. Default parameter for both the alignments was gap initial penalty- 8 and gap extension penalty of 2. The alignment was fixed under the PAM40 series protein-weight matrices in both the cases. The sequence alignments were displayed graphically using BIOEDIT package [28] with a threshold of 95% consensus residue shading.

Representative crystal structures of available cyanobacterial FeSOD (1my6-*Thermosynechococcus elongates *BP-1) and MnSOD (1gv3-*Anabaena *sp. PCC7120) with exception for NiSOD (1t6u-*Streptomyces coelicolor*) and Cu/ZnSOD (1eqw-*Salmonella typhimurium*) were retrieved from PDB. The 3D structures were analyzed using SWISS-PDB viewer [34] and graphical representations were done with WebLab viewer lite (V.4.2)

## Authors' contributions

BP and JP contributed equally in carrying out the sequence analysis studies and participated in the sequence alignment. RTD carried out further confirmation of the results and helped BP in visualization of the structures. TS helped in carrying out the structural comparison. LU and DP participated equally in the study, its design and coordination. GS helped in fine tuning of the manuscript. All authors read and approved the final manuscript written by BP.

## Supplementary Material

Additional file 1 **Excerpts of aminoacid sequences of Fe and MnSOD of cyanobacteria**. The proteins are labeled by their accession number with organism source and the metal cofactor specificity. Conserved residues for discrimination of Fe and Mn metalloforms in cyanobacteria based on multiple alignment using ClustalW of BioEdit Package (v.7.0.5) [28]. The highly conserved metal specific residues are highlighted in red for Fe and green for MnSODs. Transmembrane hydrophobic pocket specific for membrane binding in MnSOD at the N-terminal region is highlighted in violet. Residues involved in outer sphere hydrogen bonding for Mn is highlighted in cyan and for Fe in orange. For FeSOD, the lysine residues involved in photosynthetic context is shown in pink. The active site residues are marked as **I **and the dimer residues are represented by *. Click here for file
