# Supplementary figures and images for: Comparative analysis of cyanobacterial superoxide dismutases to discriminate canonical forms
Source: BMC Genomics. 2007 Nov 27;8:435. doi: 10.1186/1471-2164-8-435 (PMC2234264; doi:10.1186/1471-2164-8-435)

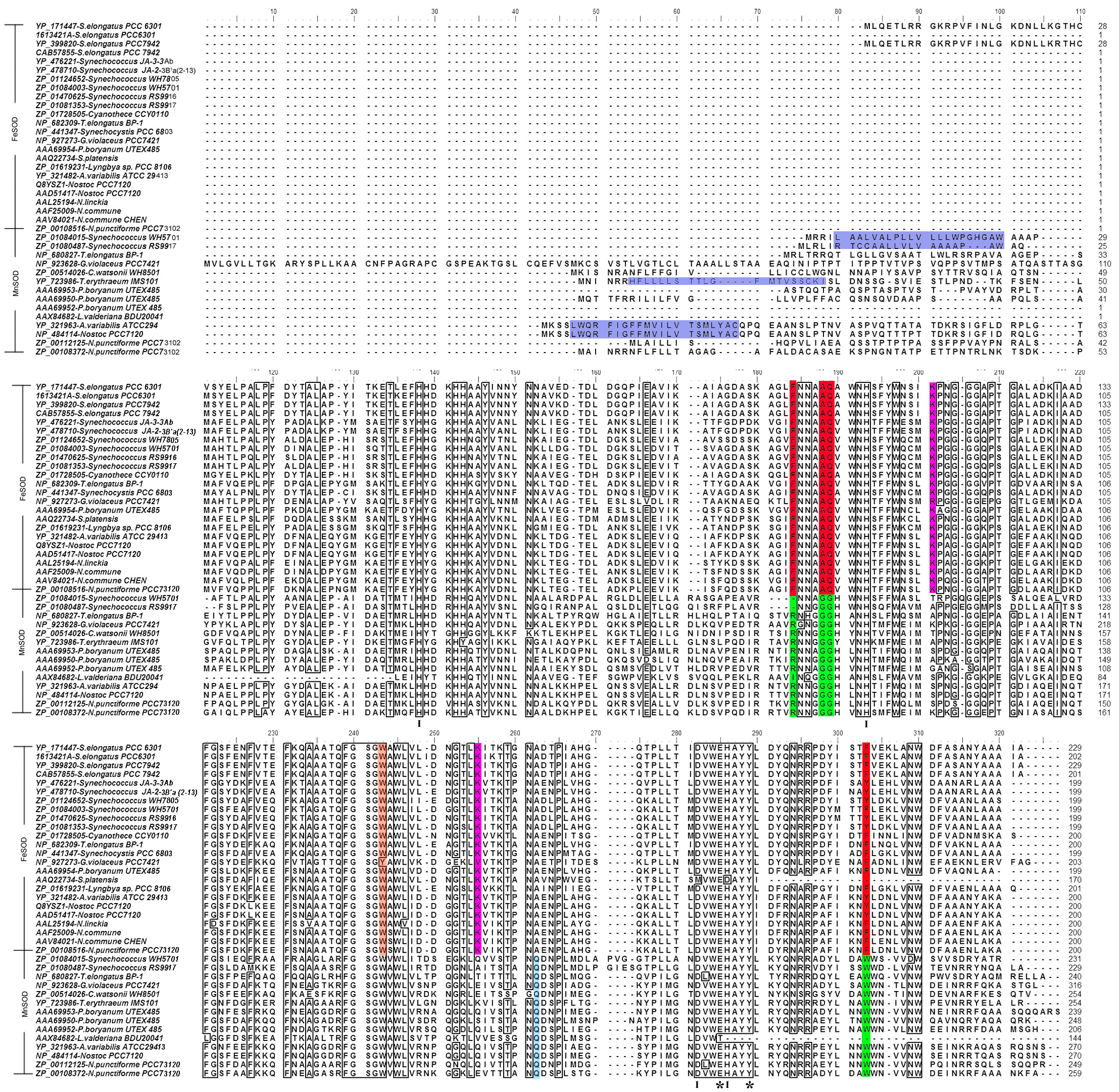

Supplement: Additional file 1 — Excerpts of aminoacid sequences of Fe and MnSOD of cyanobacteria. The proteins are labeled by their accession number with organism source and the metal cofactor specificity. Conserved residues for discrimination of Fe and Mn metalloforms in cyanobacteria based on multiple alignment using ClustalW of BioEdit Package (v.7.0.5) [28]. The highly conserved metal specific residues are highlighted in red for Fe and green for MnSODs. Transmembrane hydrophobic pocket specific for membrane binding in MnSOD at the N-terminal region is highlighted in violet. Residues involved in outer sphere hydrogen bonding for Mn is highlighted in cyan and for Fe in orange. For FeSOD, the lysine residues involved in photosynthetic context is shown in pink. The active site residues are marked as I and the dimer residues are represented by *. [file 1471-2164-8-435-S1.jpeg]
